# The androgen receptor can signal through Wnt/β-Catenin in prostate cancer cells as an adaptation mechanism to castration levels of androgens

**DOI:** 10.1186/1471-2121-9-4

**Published:** 2008-01-24

**Authors:** Liang Schweizer, Cheryl A Rizzo, Thomas E Spires, J Suso Platero, Qiuyan Wu, Tai-An Lin, Marco M Gottardis, Ricardo M Attar

**Affiliations:** 1 Oncology Drug Discovery, Pharmaceutical Research Institute, Bristol-Myers Squibb Company, Princeton, NJ, USA; 2 Clinical Discovery Technologies, Pharmaceutical Research Institute, Bristol-Myers Squibb Company, Princeton, NJ, USA; 3 Inflammation Discovery Research, Hoffmann - La Roche Inc., Nutley, NJ, USA

## Abstract

**Background:**

A crucial event in Prostate Cancer progression is the conversion from a hormone-sensitive to a hormone-refractory disease state. Correlating with this transition, androgen receptor (AR) amplification and mutations are often observed in patients failing hormonal ablation therapies. β-Catenin, an essential component of the canonical Wnt signaling pathway, was shown to be a coactivator of the AR signaling in the presence of androgens. However, it is not yet clear what effect the increased levels of the AR could have on the Wnt signaling pathway in these hormone-refractory prostate cells.

**Results:**

Transient transfections of several human prostate cancer cell lines with the AR and multiple components of the Wnt signaling pathway demonstrate that the AR overexpression can potentiate the transcriptional activities of Wnt/β-Catenin signaling. In addition, the simultaneous activation of the Wnt signaling pathway and overexpression of the AR promote prostate cancer cell growth and transformation at castration levels of androgens. Interestingly, the presence of physiological levels of androgen or other AR agonists inhibits these effects. These observations are consistent with the nuclear co-localization of the AR and β-Catenin shown by immunohistochemistry in human prostate cancer samples. Furthermore, chromatin immunoprecipitation assays showed that Wnt3A can recruit the AR to the promoter regions of Myc and Cyclin D1, which are well-characterized downstream targets of the Wnt signalling pathway. The same assays demonstrated that the AR and β-Catenin can be recruited to the promoter and enhancer regions of a known AR target gene PSA upon Wnt signaling. These results suggest that the AR is promoting Wnt signaling at the chromatin level.

**Conclusion:**

Our findings suggest that the AR signaling through the Wnt/β-Catenin pathway should be added to the well established functional interactions between both pathways. Moreover, our data show that via this interaction the AR could promote prostate cell malignancy in a ligand-independent manner.

## Background

Prostate cancer is the second leading cause of cancer-related deaths among men in the U.S., after lung cancer. Since the prostate gland is an androgen-dependent organ, prostate cancer initially responds to androgen ablation. However, this type of treatment is almost never curative, and the majority of patients will evolve from a hormone-sensitive to a lethal castration-refractory form of the disease. It has been postulated that increased levels of both the androgen receptor (AR) mRNA and protein are associated with this transition [1-3]. In addition, the AR activating mutations, as well as coactivator upregulation, were suggested to be involved in the progression of the disease to the castration-independent state [4-7]. Single amino acid substitutions in the AR ligand binding pocket such as T877A or H874Y, which change the ligand specificities of the AR, have been reported in patients with castration-resistant metastatic prostate cancer [8,9]. Major efforts have been made in prostate cancer research to understand what role the AR, either amplified wild-type or its different mutated versions, plays in these late-stage prostate cancer cells. Accumulating evidence indicates that various growth signals and cytokines, such as the insulin-like growth factor-1, the HER-2/neu tyrosine kinase and the Wnt/β-Catenin signaling pathways [10-13], can stimulate the transcriptional activity of the AR.

The Wnt family of signaling proteins plays important roles in stem cell self-renewal and multiple developmental processes. Deregulation of Wnt signaling can lead to various types of cancer [14,15]. The cytoplasmic stabilization of β-Catenin, a key component of the canonical Wnt signaling pathway, and its resulting nuclear accumulation, is a hallmark of the Wnt signaling pathway activation. In a simplified overview of the canonical Wnt/β-Catenin pathway, Wnt binds to its receptors Frizzled and LRP5/6, activating the downstream component Dishevelled, which in turn inhibits Glycogen Synthetase Kinase (GSK-3β), Axin and Adenomatous Polyposis Coli (APC) in the β-Catenin destruction complex. Once stabilized, β-Catenin binds to LEF/TCF transcription factors in the nucleus, and together they activate transcription of the targets of the Wnt signaling pathway. Stabilizing mutations of β-Catenin and increased levels of nuclear β-Catenin have been reported in castration-resistant PCa (recently reviewed by Yardy and Brewster [16]). It has been shown that β-Catenin interacts with the AR and potentiates the AR signaling in an androgen-dependent fashion in prostate cells [17-20]. Therefore, it was suggested that β-Catenin exerts its cancer-related function, in part, through the AR signaling [13]. On the other hand, the AR signaling was shown to repress β-Catenin/TCF mediated transcription induced by androgen in prostate cancer cells [20-22]. However, the relationship between the AR and β-Catenin has not been examined in prostate cancer cells exposed to castration levels of androgens. Recently, TCF4 and GSK-3β were also shown to interact with the AR in mediating the AR activity [23]. Therefore, the interaction between the AR and the entire Wnt signaling pathway needs to be further explored in order to characterize possible tumor refractory mechanisms in castration-resistant prostate cancer cells.

Here we show that the AR can potentiate Wnt signaling in prostate cancer cells. The interaction between the AR and Wnt signaling provides a growth advantage to prostate cancer cells at castration levels of androgens. Interestingly, we found that the presence of the AR ligands attenuated the input of the AR into the Wnt signaling pathway. Physiological levels of androgens inhibited the tumor cell growth and transformation mediated by the activation of the Wnt signaling and the AR overexpression. These results are in agreement with the observation that the AR and β-Catenin can co-localize in the nucleus of human prostate cancer cells. To further explore the mechanism of the AR and Wnt interaction, we have demonstrated by the use of Chromatin Immunoprecipitation (ChIP), that Wnt can recruit the AR and β-Catenin onto the promoter regions of the Wnt target genes *myc *and *cyclin D1*, as well as to the promoter and enhancer regions of the androgen target gene PSA. These ChIP results indicate that the AR potentiates the Wnt signal at the transcriptional level. In sum, our finding provides a novel mechanism of the AR function, namely, promoting Wnt/β-Catenin signaling and its resulting oncogenic properties in prostate cancer cells at castration levels of androgens.

## Results

### AR overexpression potentiates Wnt/β-Catenin transcriptional activities

**Figure 1:**
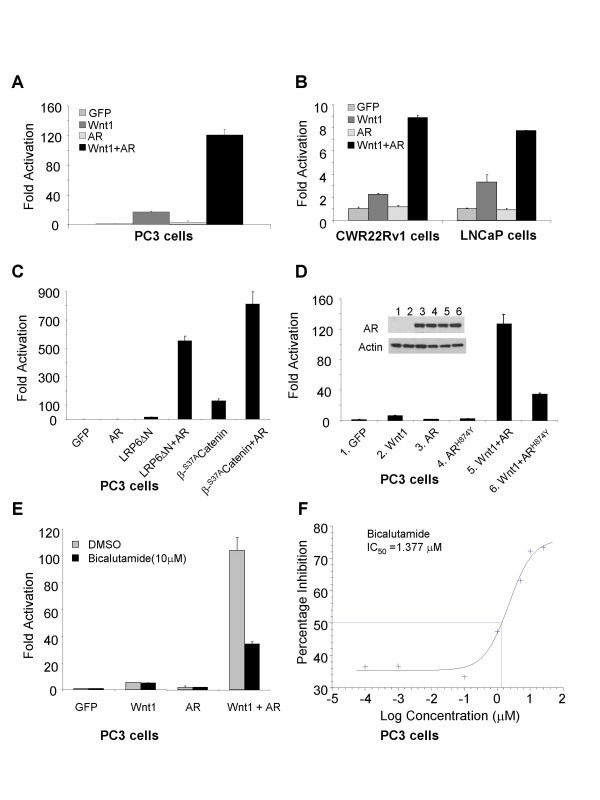
**The overexpression of wild-type or mutant AR potentiates Wnt signaling**. Cells grown in RPMI with 10% FBS were transiently transfected with indicated plasmids together with LEF-luciferase and pCMV-Renilla reporter constructs in 12 well plates. Cotransfection of Wnt1 and AR leads to a synergistic effect on the activation of LEF-luciferase reporter in PC3 cells (A), CWR22Rv1 and LNCaP cells (B), compared with transfection with Wnt1 alone. The AR can also potentiate the activation of Wnt signaling in cells expressing a constitutively activated human receptor LRP6, truncated at the N terminus (H6ΔN), or a stabilized β-Catenin ^S37A ^(C). The synergistic effect between AR mutants and Wnt1 signaling is shown (D). AR expression levels were measured for both wild type and mutant AR^H874Y^. Actin levels were used as loading controls. The effect of an AR antagonist is shown (E & F). PC3 cells were treated with bicalutamide 4 hours after transfection. Dual luciferase assay was performed 20–24 hours after treatment.

While the role of activated Wnt/β-Catenin signaling in prostate cancer is not entirely clear, recent evidence indicates that β-Catenin contributes to prostate cancer progression through its enhancement of the AR transcriptional activity in the presence of androgens [13]. Reciprocally, using transient transfections of prostate cancer cells lines, we explored whether the AR overexpression had any effect on the Wnt/β-Catenin signaling pathway. As expected, expression of Wnt1 in the prostate cancer cells PC3 (Figure 1A), CWR22Rv1 and LNCaP (Figure 1B) resulted in the activation of the luciferase reporter gene driven by a LEF-dependent promoter [24]. This activation ranged from 2 to 20 fold compared with control cells transfected with GFP, depending on the cell type and assay conditions. The expression of the AR alone had little effect on LEF-dependent transcription in these cells under these assay conditions, although the transfection of relatively high amounts of the AR did lead to a 2–5 fold activation of the reporter gene (data not shown). Interestingly, the co-expression of Wnt1 and AR resulted in increased transactivation, compared to the effect of Wnt1 alone (7-fold in PC3, 4-fold in CWR22Rv1, and 2.5-fold in LNCaP cells) (Figure 1A &1B). This effect is more dramatic in PC3 cells than in CWR22Rv1 and LNCaP cells, probably due to the fact that PC3 cells have no detectable levels of the AR expression, while both CWR22Rv1 and LNCaP cells express endogenous mutant forms of the AR. We also tested the ability of the AR to potentiate other signaling components of the Wnt signaling pathway, such as the constitutively activated receptor LRP6 with a truncated N terminus (LRP6ΔN) [24], or the stabilized β-Catenin mutant (β-^S37A^Catenin) [25]. While the activation of the Wnt/β-Catenin signaling pathway in PC3 cells transfected with LRP6ΔN led to a 12-fold increase in the luciferase activity compared with the GFP control, the co-expression of the AR increased the activity 46-fold compared with LRP6ΔN signaling alone (Figure 1C). Cotransfection of β-^S37A^Catenin together with the AR resulted in a 6-fold activity increase in LEF mediated transcription compared with β-^S37A^Catenin activity alone (Figure 1C). Similar effects were also observed when the Wnt signaling pathway was activated by co-expression of Dishevelled or Casein Kinase I with the AR in PC3 cells. In this case, the AR enhanced the signaling activity of Dishevelled and Casein Kinase I by 6- and 10-fold respectively (data not shown). Based on these results, we conclude that the AR can potentiate Wnt transcriptional activity in prostate cancer cells.

Since CWR22Rv1 cells express mutant forms of AR^H874Y ^[26], and the LNCaP cells express AR^T877A ^[27], we explored whether those mutant forms of the AR could also stimulate Wnt signaling. Wnt1 together with either wild-type or mutant AR expression constructs were cotransfected into PC3 cells. Indeed, the mutant AR^H874Y ^also enhanced Wnt1 signaling with at least 5-fold increases when compared to Wnt1 activity alone, although this activity was reduced compared with that of the wild-type AR (Figure 1D). To prove that the difference in activity was not due to difference in expression, the levels of AR and AR^H874Y ^were examined. As shown in Figure 1D, the expressions of both forms of AR were equivalent. Similar results were obtained for the AR^T877A ^mutation as well (data not shown). These results indicate that the mutated AR expression in CWR22Rv1 and LNCaP cells can potentiate Wnt signaling.

To confirm our observation that the AR potentiates Wnt signaling, bicalutamide was used to determine whether an AR antagonist could inhibit this potentiation. At concentrations as high as 10 μM, bicalutamide did not have any effect on the transcriptional activities of the LEF-dependent luciferase reporter in PC3 cells when cells were transfected with Wnt1 or AR alone. However, it inhibited approximately 60% of the transcriptional activity promoted by the co-expression of the AR and Wnt1 (Figure 1E), suggesting that when bound to an antiandrogen, the AR is less capable of potentiating Wnt signaling. Noticeably, this inhibition was observed in cells cultured with 5% charcoal-stripped serum without the addition of an AR agonist such as 5α-dihydrotestosterone (DHT). This indicates the mechanism by which bicalutamide inhibits the synergy between Wnt and AR is not likely to be the displacement of an agonist, but rather through the binding of bicalutamide to the AR. To characterize this inhibition further, bicalutamide was tested in a dose-dependent manner with concentrations ranging from 0.1 nM to 25 μM in the LEF-dependent luciferase reporter assay, using PC3 cells co-transfected with both Wnt1 and AR. Bicalutamide showed an IC_50 _of 1.38 μM in this study (Figure 1F).

### AR interacts with Wnt signaling to promote prostate cancer cell proliferation

**Figure 2:**
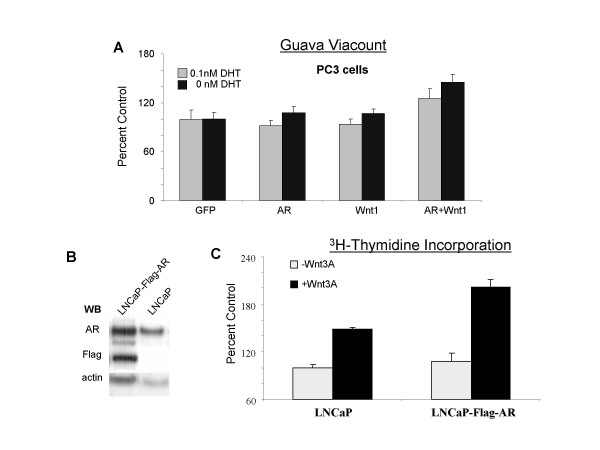
**The synergy of Wnt and AR promotes proliferation in prostate cancer cells**. (A) PC3 cells in phenol-red free RPMI with 5% charcoal-stripped serum were transfected with the indicated plasmids and treatments in 96 well plates. 7 days after transfection, the number of cells was determined with a Guava proliferation assay. Cells transfected with GFP were used as control. The percentage of cell numbers was calculated for cells transfected with Wnt or AR or both compared with control samples. (B) AR expression and Flag-tagged AR in LNCaP-Flag-AR cells and LNCaP cells were detected by Western blot. Actin was used as loading control. (C). LNCaP cells and LNCaP-Flag-AR cells in phenol-red free RPMI with 5% charcoal-stripped serum were treated with 100 ng/ml Wnt3A for four days in 96 well plates. The cells were then labelled with [^3^H]-thymidine and harvested to measure the amount of thymidine incorporation. LNCaP cells which received no Wnt3A treatment were used as control. The percentage of [^3^H]-thymidine incorporation was calculated compared with control.

To investigate whether the synergy between the AR and Wnt transcriptional activities would lead to accelerated tumor cell growth, we set out to measure the growth of PC3 cells under the conditions of overexpressing the AR alone, Wnt1 alone, or the combination of both. The transfection efficiency was monitored by the expression of a control GFP. Around 90% of the PC3 cells had green fluorescence under a fluorescent microscope, indicating a high level of transfection in those cells. Cell numbers were then obtained after seven days post-transfections and incubation in the absence or presence of 0.1 nM DHT using Guava technology. As shown in Figure 2A, cells transfected with either Wnt1 or AR alone do not show significant differences in cell numbers compared with control cells transfected with GFP (p < 0.2) in the absence or presence of 0.1 nM DHT. However, cotransfection of Wnt1 and AR increased the number of PC3 cells significantly compared with either one of them alone: with AR (p < 0.005), or with Wnt1 (p < 0.007) in the absence of DHT; and with AR alone (p < 0.027), or with Wnt alone (p < 0.003) in the presence of 0.1 nM DHT. These results suggest that the interaction between these two signaling pathways at castration levels of androgens promotes prostate cancer cell growth more significantly compared with the activation of either pathway alone.

We further confirmed this growth stimulation effect in the parental LNCaP cell line, which expresses an endogenous AR^T877A^, as well as in an engineered LNCaP-Flag-AR cell line that also stably expresses a Flag-tagged wild-type AR (Figure 2B). Since the efficiency of transfection is usually less than 50% in these cells compared with around 90% in PC3 cells, we used commercially-available recombinant Wnt3A in this set of experiments to ensure the exposure of Wnt ligand to all cells. In addition, since these cells tend to cluster, the assessment of proliferation was carried out by measuring the incorporation of [^3^H]-thymidine rather than counting cells directly. As expected, LNCaP-Flag-AR cells proliferated faster than LNCaP cells in both the presence and the absence of Wnt3A treatment because of the wild-type AR overexpression. As shown in Figure 2C, the treatment of LNCaP cells with Wnt3A increased cell proliferation up to 50%, possibly due to the interaction between the endogenous mutant AR and the Wnt3A signaling. Furthermore, in the LNCaP-Flag-AR cells, Wnt3A dramatically increased the cell proliferation by nearly 100% due to the overexpression of the wild-type AR. The cells shown in 2C were cultured in charcoal-stripped serum with no addition of DHT. Similar results were also obtained in cells exposed to low levels of androgen (such as 0.01 nM and 0.1 nM DHT, data not shown). Taken together, these results suggest that at castration levels of androgens, the amplified wild-type or mutant AR can synergistically interact with the Wnt signaling pathway, resulting in the stimulation of prostate cancer cell growth.

### AR agonists inhibit the AR potentiation of the Wnt signaling pathway

**Figure 3:**
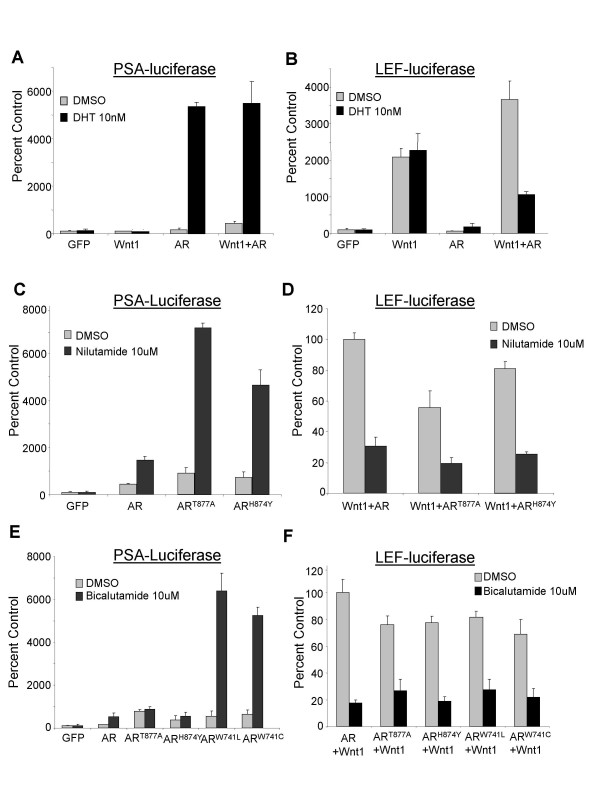
**AR agonists inhibit the Wnt and AR synergy**. PC3 cells in phenol-red free RPMI with 5% charcoal-stripped serum in 96 well plates were transfected with the indicated plasmids, together with pCMV-Renilla reporter and PSA-luciferase (A, C, & E) or LEF-Luciferase (B, D, & F). The following treatments were applied to cells 4 hours after transfection: 10 nM DHT (A & B); 10 uM Nilutamide (C & D); 10 uM Bicalutamide (E & F). Dual luciferase assays were performed 20–24 hours after treatment.

We next examined the effect of androgens on the transcriptional synergy between Wnt1 and the AR. As expected, 10 nM DHT stimulated the AR signaling pathway, inducing the expression of the luciferase reporter gene driven by the PSA promoter in PC3 cells transfected with either the AR, or with both the AR and Wnt (Figure 3A). Interestingly, 10 nM DHT stimulated PSA-luciferase activity similarly in cells transfected with the AR alone or with both the AR and Wnt1 (Figure 3A), implying that additional β-Catenin did not necessarily translate into further activation of the AR signaling pathway. On the other hand, 10 nM DHT inhibited approximately 70% of the expression of the LEF-driven luciferase reporter in PC3 cells co-transfected with Wnt and AR (Figure 3B). However, it had no inhibitory effect when cells were transfected with Wnt1 alone (Figure 3B), indicating that similar to bicalutamide, its mechanism of action is through binding to the AR. We also noticed that DHT not only abrogated the AR-Wnt synergy, but also reduced the LEF-dependent transcription to levels lower than what was observed in cells transfected only with Wnt 1 (Figure 3B). This may be due to the fact that DHT can recruit β-Catenin into the AR signaling pathway, an observation that has been confirmed by other groups [17-20].

In order to confirm that the inhibition of the AR-Wnt potentiation by any AR ligand is independent of its pharmacological activity, we explored the effect of nilutamide, a small non-steroidal molecule that is an agonist of the mutant AR^T877A ^expressed in LNCaP cells and possibly also an agonist of the mutant AR^H874Y ^found in the CWR22 xenograft model [27-30]. As shown in Figure 3C, nilutamide stimulated the PSA promoter-driven transcription of the luciferase reporter when cotransfected with either the AR^T877A ^or the AR^H874Y ^mutants into PC3 cells. However, it had less of an effect on the wild-type AR-mediated transcription. This result is consistent with previous reports [27-30]. In addition, we observed that nilutamide impeded at least 60% of the LEF-dependent transcription activity in PC3 cells cotransfected with Wnt1 and either wild-type AR, AR^T877A^, or AR^H874Y ^(Figure 3D). Recently, bicalutamide was identified as an agonist for the AR^W741L ^and AR^W741C ^mutations [31]. This effect was confirmed in our transactivation assays performed in PC3 cells cotransfected with the mutant AR expression plasmids and the PSA promoter-driven luciferase reporter construct. Bicalutamide indeed stimulated the transcriptional activity through these two particular AR mutants, but not through the wild-type AR or the LNCaP or CWR22 AR variants (Figure 3E). Bicalutamide also repressed the synergy between Wnt1 and either the wild type AR or any of the mutant AR isoforms in PC3 cells (Figure 3F). This set of experiments demonstrates that AR ligands, agonists, or antagonists can prevent the AR synergy with the Wnt signaling pathway.

**Figure 4:**
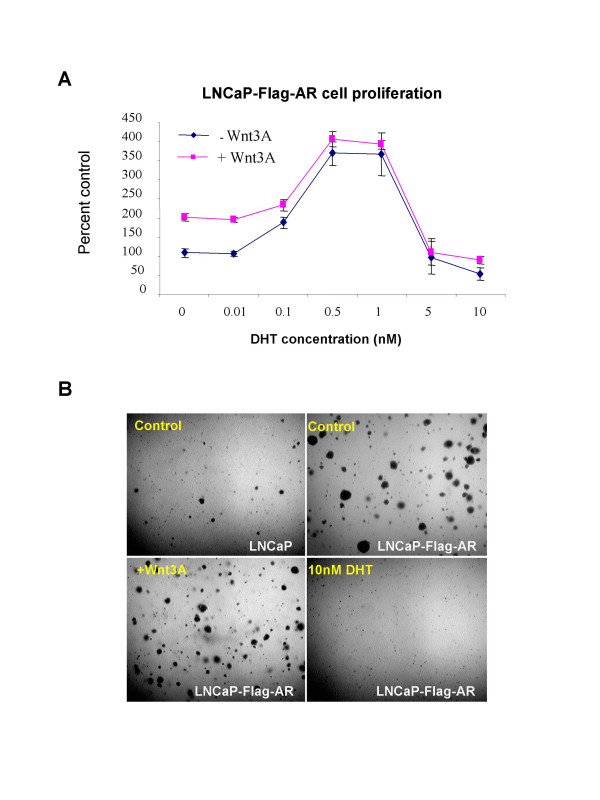
**The effect of DHT on cell proliferation and soft agar growth**. (A) LNCaP-Flag-AR cells in phenol-red free RPMI with 5% charcoal-stripped serum were treated with DHT ranging from 0.01 nM to 10 nM with or without Wnt3A 100 ng/ml for four days in 96 well plates. The cells were then labeled with [^3^H]-thymidine and harvested to measure the thymidine incorporation. Cells that received no DHT treatment were used as control. The percentage of [^3^H]-thymidine incorporation was calculated compared with control. (B). LNCaP and LNCaP-Flag-AR cells were plated in soft agar with no treatment as control or with 100 ng/ml Wnt3A, or 10 nM DHT. After approximately 4 weeks, colonies were fixed with 10% formaldehyde in PBS. A representative field of cells was photographed for each cell type, with or without treatment using bright-field microscopy. Upper panel LNCaP or LNCaP-Flag-AR cells received no treatment, while lower panel, LNCaP-Flag-AR cells were either treated with Wnt3A or DHT.

To further examine the effects of androgens on prostate cancer growth mediated by the Wnt and AR signaling, we took advantage of the stable LNCaP-Flag-AR cell line that overexpresses the wild-type AR (Figure 2B) mimicking the hormone -refractory state [3]. We examined if this cell line behaves differently from its parental LNCaP cell line in the presence or absence of Wnt3A treatment. Cell proliferation was assessed by measuring [^3^H]-thymidine incorporation. Similar to LNCaP cells [32], LNCaP-Flag-AR cells showed a biphasic response to DHT treatments (Figure 4A). Wnt3A stimulated LNCaP-Flag-AR cell growth noticeably by 2 fold with 0.01 nM DHT or no DHT treatment. The maximum increase of LNCaP-Flag-AR cell growth by DHT at 0.5 nM or 1 nM concentration was 4-fold. Higher concentrations of DHT at 5 nM and 10 nM reduced cell proliferation by approximately 50% either with or without Wnt3A treatment (Figure 4A). These results indicate that normal physiological levels of serum DHT concentration may inhibit cell proliferation driven by the AR overexpression and Wnt signaling.

We further confirmed our observation by performing soft agar growth assays to measure anchorage-independent growth of LNCaP and LNCaP-Flag-AR cells treated either with Wnt3A or with DHT. As seen in Figure 4B, LNCaP-Flag-AR showed an increased soft agar growth compared with the parental LNCaP cell line. This agrees with past reports that the AR overexpression increased the oncogenic malignancy and transformation of prostate cancer cells. Wnt3A treatment appeared to stimulate colony numbers on soft agar, but not the colony size. Similarly, as observed with the cell proliferation assay by [^3^H]-thymidine incorporation in LNCaP-Flag-AR cells, 10 nM DHT treatment did not stimulate, but rather reduced the soft agar growth of the LNCaP-Flag-AR cells. When these cells were treated both with Wnt3A and 10 nM DHT, the results were similar to that of 10 nM DHT alone (data not shown). These results suggest that the restoration to physiological levels of androgens could decrease the malignant tendency of castration-refractory prostate cancer cells, possibly by inhibiting the input of the AR into the Wnt/β-Catenin signaling pathway.

### AR is promoting Wnt signaling at the chromatin level

**Figure 5:**
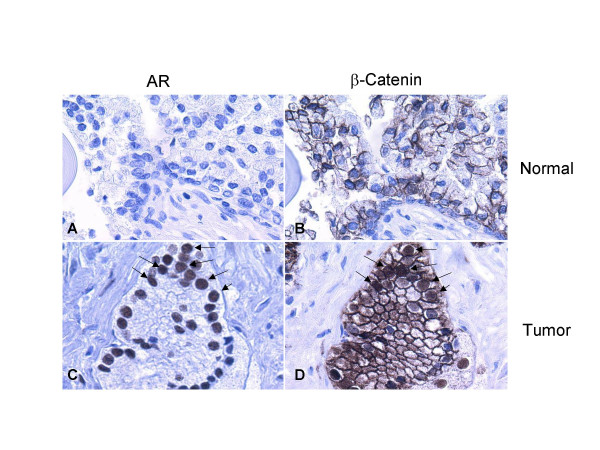
**Expression of AR and β-Catenin in normal prostate tissues and tumor samples**. A tissue microarray containing 9 prostate tumors and 4 normal prostate tissue samples was stained with antibodies against AR and β-Catenin. In normal prostate tissues, the low levels of AR expression, compared with the high levels in prostate cancer cells, were indicated by lack of AR staining in cells (A); β-Catenin was predominantly located at the normal cytoplasmic membrane (B). In late stage prostate cancer samples, AR was substantially overexpressed in the nuclei of the prostate cancer cells (C) where nuclear β-Catenin staining was also observed in some of these cells (D).

Since both the AR and β-Catenin translocate into the nucleus upon androgen and Wnt signaling respectively, we performed immunohistochemistry staining using a tissue microarray with the AR and β-Catenin specific antibodies to explore the possible co-localization of the AR and β-Catenin in human prostate cancer samples. Four normal prostate tissue samples and nine prostate tumors were included in this tissue microarray. In the tissue array tested, the AR expressed at a much higher level in the tumor samples compared with the normal prostate tissues. In order to observe clear nuclear staining in the tumor samples, we calibrated the staining to conditions that gave a very low or negative signal in normal prostate tissues (Figure 5A). Those conditions revealed a strong nuclear AR staining in prostate cancer cells with Gleason Score (GS) 6 (Figure 5C) and above. These studies demonstrated a considerable AR staining in the nucleus of the nine tumors. In the meantime, β-Catenin was localized to the cell membrane in all four normal prostate tissue samples (Figure 5B), whereas we observed nuclear β-Catenin staining in three out of nine prostate tumors from patients with Gleason Score 6 (Figure 5D) and above. Since the sections of these stainings are consecutive, it is possible to identify the same cells with both AR and β-Catenin nuclear staining (see arrows). These results are not only consistent with the reports that associate high levels of Wnt-1 and β-Catenin expression with advanced metastatic, hormone-refractory prostate carcinoma [33,34], but they also agree with the suggestion of a functional interaction between the AR and β-Catenin upon co-localization in the nucleus of prostate cancer cells at late stages.

**Figure 6:**
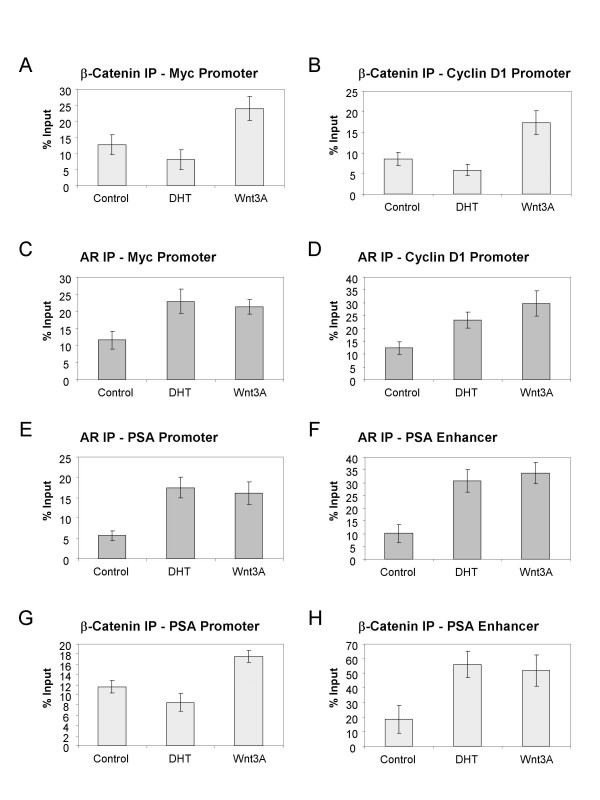
**The recruitment of AR and β-Catenin to Wnt signaling target genes as well as PSA promoter and enhancer region using CHIP analysis**. LNCaP cells in phenol-red free RPMI with 5% charcoal-stripped serum were treated with either 10 nM DHT, 100 ng/ml Wnt3A, or no treatment for 16 hours before cross-linking. Anti-β-Catenin (A, B, G and H) and anti-AR (C, D, E and F) antibodies, together with negative control IgG were then used for immunoprecipitation. After reverse crosslinking and DNA purification, PCR products were analyzed using the Agilent 2100 bioanalyzer. Percent of input is shown here to compare the levels of β-Catenin or AR at the promoter or enhancer region of PSA, or the promoter region of Cyclin D1 or Myc.

To investigate further the interaction between the AR and β-Catenin in the nucleus, we examined through chromatin immunoprecipitation assays (ChIP), whether the AR can be recruited to the promoter region of Wnt target genes in LNCaP cells. Since it has been shown that Wnt signaling can activate both Myc and Cyclin D1, which contain LEF/TCF binding sites in their promoter regions [35,36], we asked if Wnt signaling could bring the AR to these regions as well. Indeed, Wnt3A stabilized β-Catenin and increased its binding to the *myc *and *cyclin D1 *promoter regions (Figure 6A, 6B). In addition, the treatment of LNCaP cells with Wnt3A resulted in the recruitment of the AR to the promoter regions of both *myc *and *cyclin D1 *(Figure 6C, 6D), implying a direct transcriptional involvement of the AR at the promoters of these Wnt target genes upon activation of Wnt signaling. Interestingly, LNCaP cells treated with a physiological level of DHT (10 nM) might have reduced amounts of β-Catenin at the *myc *and *cyclin D1 *promoters compared with untreated samples (Figure 6A, 6B), possibly due to the fact that cell stimulation with DHT causes recruitment of β-Catenin to the promoter and enhancer regions of PSA (Figure 6E, 6F). We also found that DHT recruited the AR to the *myc *promoter and possibly the *cyclin D1 *promoter in the absence of Wnt signaling (Figure 6B, 6C), indicating that direct AR binding sites may be present in these promoter regions. This finding is in agreement with the report that endogenous AR was bound to a TCF-4 responsive element in the c-Myc promoter [37].

We also examined the promoter and enhancer regions of PSA, a well characterized AR regulated target gene. As reported [38], DHT treatment promoted the binding of the AR at both of these regions (Figure 6E, 6F). Interestingly, in the absence of androgens the AR was also recruited to the PSA promoter and enhancer regions as a result of Wnt3A signaling (Figure 6E, 6F). This suggests that upon Wnt stimulation, the stabilized β-Catenin could bind to the AR, leading to its signaling activation. This was further confirmed when β-Catenin was found at the promoter and enhancer regions of PSA upon treatment with Wnt3A (Figure 6G, 6H). These ChIP results demonstrate a novel mechanism by which Wnt stimulates the transcriptional activity of the AR in the absence of androgens.

## Discussion

The androgen receptor signaling pathway has been shown to play an essential role in castration-refractory prostate cancer [2,3,39,40]. Recent emerging evidence indicates that the Wnt/β-Catenin signaling pathway is involved in advanced prostate cancer [13,33,34,41,42], and it is known that β-Catenin can signal through the AR in the presence of androgens. We determined the influence of the AR on the Wnt/β-Catenin transcriptional activity in prostate cancer cells with activated Wnt signaling under conditions that mimic the castration-resistant stage. It was reported that the mean total serum testosterone concentration and the androgen bioactivity in the serum of normal men were from 8 nM to 12 nM [43], while the castration level is below 3 nM [44]. The current study was conducted using concentrations of androgens similar to the castration levels in several prostate cancer cell lines. Our results demonstrated a novel mechanism for the AR function by potentiating the Wnt signaling pathway to promote prostate cancer cell growth at the castration levels of androgens. These results are not only consistent with the report by Verras *et al*. [42] that Wnt3A conditional medium promotes LNCaP cell growth both in ligand-dependent and independent manners, but they also agree with the observation that Wnt3a stimulated proliferation selectively in the AR positive prostate cancer cells, but not in AR negative cells [45]. We further observed that mutant forms of AR have a similar or possibly reduced ability to stimulate Wnt1/β-Catenin signaling. This finding could offer an explanation for the high incidence of wild-type AR overexpression observed in advanced castration-refractory prostate cancer specimens. Our results demonstrated that both AR agonists and antagonists can inhibit the positive effects of both the wild-type and mutant AR isoforms on the Wnt signaling pathway. This intriguing phenomenon might suggest that the ligand-bound AR leads to interactions with other cofactors in the AR signaling pathway[46], therefore reducing the ability of AR to signal into the Wnt/β-Catenin pathway. One such complex that involves the AR and its ligands, regardless of their agonistic or antagonistic nature, is the interaction with heat shock proteins. Upon ligand binding, the AR is dissociated from the heat shock complex and binds to DNA after undergoing dimerization. We can hypothesize that the potentiation of the AR on the Wnt/β-Catenin signaling pathway requires the presence of components of the heat shock protein complex, or alternatively that it requires the AR in its monomeric form in order to complex with β-Catenin as a heterodimer. This could explain why both the AR agonists and antagonists inhibit the AR input into the Wnt signaling pathway. In this report, we demonstrated that physiological levels of DHT reduced the malignancy of LNCaP cells with the AR overexpression. This might provide a possible mechanism for the potential therapeutic benefit of intermittent androgen suppression. Finally, our mechanistic study showed that Wnt can signal through the binding of the AR and β-Catenin at the PSA promoter and enhancer regions, indicating that Wnt-stabilized β-Catenin can promote AR transcriptional activities under the conditions of androgen ablation. This interesting observation is being further explored. Since it has been shown that TCF-4 interacts with the AR directly and that the interaction might occur on the promoters or enhancers of certain genes [37], the role of TCF4 in this interaction, as well as the identification of the domains of the AR and beta-Catenin involved, is being investigated.

Previous studies showed that TCF target gene transcription can be suppressed by AR in a ligand-dependent manner [20-22]. While two such reports used cell lines other than prostate cells for their investigation, the work by Chesire and Isaacs was conducted in a variety of prostate cancer cells. It is interesting to note that in LAPC-4 cells that express endogenous wild-type AR, the addition of increased amounts of androgen did not show any inhibition on the TCF transcriptional reporter activated by overexpression of a stabilized β-Catenin mutant. Furthermore, when DU145 cells were transfected with the wild-type AR or mutant AR with deleted DNA binding domain (DBD), an increase of TCF transcriptional activities was observed [19]. In addition, overexpression of this mutant did not lead to ligand-dependent interference of TCF transcription. These observations are in agreement with what we have observed in our study. Another aspect that needs to be considered when comparing our findings with previous ones is that many of those studies used the β-Catenin stabilized mutants for the activation of the Wnt signaling pathway. The level of signaling activation is often very high using β-Catenin stabilized mutants, and therefore, it is difficult to observe any synergy between the β-Catenin stabilized mutants and any other positive regulator of the pathway. Our study used several positive signaling components of the Wnt signaling pathway, such as Wnt1, truncated LRP6, Dishevelled etc., and the AR potentiation effects on the luciferase reporter are consistent among those signaling components. Very recently, it has been reported that AR protein expression was down-regulated in the presence of Wnt ligand, although the AR mRNA level was increased by Wnt signaling [47]. In our study we have observed that high levels of nuclear AR staining and β-Catenin staining may co-localize in prostate cancer cells. This indicates that the mechanism of AR protein down-regulation by Wnt signaling may not exist in all prostate cancer cells.

**Figure 7:**
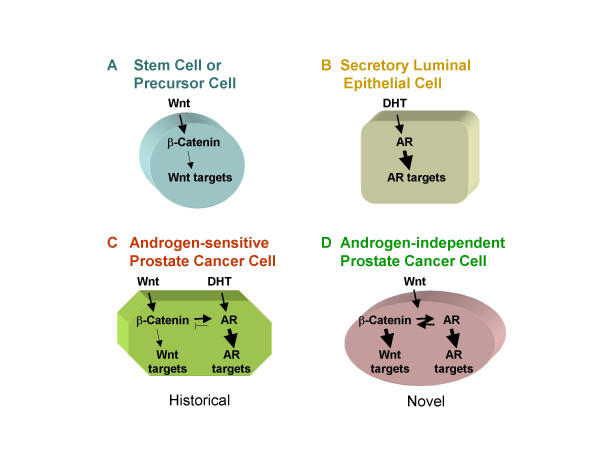
**Working model for the interaction of AR and Wnt/β-Catenin signaling pathway in castration-resistant prostate cancer cells**. In normal conditions, Wnt and androgen signaling function at different cell populations (A&B). In prostate cancer where both pathways may be deregulated in the same cells, androgens may inhibit the input of AR into the Wnt signaling pathway where β-Catenin can potentiate the AR signaling (C). In castration-resistant prostate cancer cells, Wnt can activate both signaling pathways turning the unliganded AR into a coactivator of the Wnt dependent transcriptional program (D).

To reach a full understanding of the Wnt/β-Catenin pathway in prostate cancer, a better comprehension of the specific role that the Wnt/β-Catenin signaling pathway plays in normal prostate tissue is required. The Wnt/β-Catenin pathway has been implicated in the maintenance of stem or progenitor cells in adult tissues such as blood, intestine, muscle and mammary glands [48-53]. It was suggested that a major aspect of β-Catenin signaling in normal prostate physiology is to renew the precursor cells in the basal compartment of gland acini [13]. The AR is known to be expressed in differentiated luminal glandular epithelial cells [54]. The distinct expression patterns indicate that these two signaling pathways may normally function in different cell populations (Figure 7A and 7B). In hormone-sensitive prostate cancer cells, the androgen-bound AR can inhibit the Wnt/β-Catenin signaling [19] (Figure 7C). However, in prostate tumor cells, especially in those that have adapted to growth in a low androgen environment, these two signaling pathways could be found aberrantly activated, as observed in our studies. We propose that in castration-refractory prostate cancer cells, the colocalization of both AR and β-Catenin enables the AR to signal through the Wnt/β-Catenin signaling pathway, leading to a propagation of the already accelerated cell growth and an increased state of malignancy compared with the cells that only have one of the two aberrant signaling pathways (Figure 7D).

## Conclusion

Since it has been shown that androgen-independent prostate cancer is heterogeneous and multifocal [55,56], it is possible that castration-refractory tumors derive from different combinations of the AR amplification, AR mutations and deregulated signaling pathways. Our data indicates that in hormone-refractory prostate cancer cells, the AR signals through the Wnt/β-Catenin pathway to promote tumor cell malignancy in a ligand-independent manner. Therapies targeting the input of the AR into the Wnt signaling pathway may lead to effective treatments for castration-refractory prostate tumors. Although both the standard anti-androgens and the androgens tested in this study can inhibit the input of the AR into the Wnt/β-Catenin signaling pathway, they can only reduce it to certain extent without totally abolishing it. It would be conceivable that a new generation of the AR antagonists could not only repress the AR mediated transcription, but also effectively disrupt the interaction with other pathways that can lead to the growth and transformation of castration-refractory prostate cancer.

## Methods

### Cell culture and transfection

Prostate cancer cells (PC3, CWR22Rv1 and LNCaP) were grown in RPMI 1640 medium (Invitrogen) supplemented with 10% FBS (Invitrogen) and 1% penicillin/streptomycin (Invitrogen) at 37°C in 5% CO_2_. Cells were plated into 12 well plates or 96 well plates 16 hours before transfection either in growth medium or, if not specified in the figure legend, in phenol-red-free RPMI (Invitrogen) with 5% charcoal stripped serum (Hyclone) and 1% penicillin/streptomycin. Transfections were performed with Fugene 6 (Roche) according to the manufacturer's protocol. For 12 well assays, each well received 0.02 μg of pCMV-LEF-1, 0.1 μg of LEF-luciferase reporter plasmid, and 0.01 μg pCMV-Renilla together with 0.5 μg each of the expression plasmids [24]. For 96 well assays, each well received 2 ng of pCMV-Renilla, 10 ng each expression plasmids, 4 ng of pCMV-LEF-1 and either 20 ng of the LEF-luciferase reporter or 35 ng of the PSA-luciferase reporter plasmid.

### Generation of AR Mutants

From a pCMV-Flag vector containing wild type AR, a 1507 bp fragment was excised utilizing the endogenous BstEII and SalI restriction sites. This fragment encompasses a region of the AR gene containing the three mutation sites. Using the primers listed below and the QuickChange II Site-Directed Mutagenesis kit (Stratagene), an appropriate single base pair substitution was generated for each of the mutants:

H874Y (CAT to TAT):

5' – TTGCGAGAGAGCTGTATCAGTTCACTTTTG – 3' and

5' – CAAAAGTGAACTGATACAGCTCTCTCGCAA – 3'

T877A (ACT TO GCT):

5' – AGCTGCATCAGTTCGCTTTTGACCTGCTAA – 3' and

5' – TTAGCAGGTCAAAAGCGAACTGATGCAGCT – 3'

W741L (TGG to TTG):

5' – ACCATGAGCCCCATCAAGGAGTACTGAAT – 3' and

5' – ATTCAGTACTCCTTGATGGGGCTCATGGT – 3'

W71C (TGG to TGT):

5' – ACCATGAGCCCCATACAGGAGTACTGAAT – 3' and

5' – ATTCAGTACTCCTGTATGGGGCTCATGGT – 3'

These mutant fragments were then cloned into the pCMV-Flag-AR vector lacking the corresponding 1507 bp wild type AR region. All of the mutant constructs were verified by DNA sequencing prior to these studies.

### Luciferase assays

Luciferase assays were performed with the dual luciferase assay kit (Promega) according to the manufacturer's instructions. The luciferase activities were normalized with the *Renilla *luciferase activities. The activity of control GFP expression was set to 1, and the relative activities to GFP were then calculated for fold activation. Each experiment was carried out in triplicate with error bars representing standard deviation. All the experiments were repeated three times.

### Cell growth assay

Guava Viacount: PC3 cells were plated into a 96 well plate at 1 × 10^4 ^cells per well in 100 μl phenol-red free RPMI with 5% charcoal stripped serum and 1% penicillin/streptomycin. After 24 hours, cells in each well were transfected with 100 ng of the indicated plasmids. To ensure transfection with equal amount of DNA, 100 ng of GFP was cotransfected into each well containing cells receiving only 100 ng of a single plasmid. Seven days after transfection, cells were trypsinized and stained with Guava PCA-96 Viacount reagent (Guava technologies) as indicated by the manufacturer. Cells were then transferred to a low-attachment 96 well plate and analyzed by Guava PCA-96 using the Guava ViaCount program. Each experiment was carried out in triplicate with error bars representing standard deviation.

[^3^H]-thymidine incorporation assay: LNCaP cells and LNCaP-Flag-AR cells were seeded at 6000/well in 96 well plates and maintained in phenol-red free RPMI medium supplemented with 5% charcoal-stripped serum. After 24 hours, cells were treated with either 100 ng/ml Wnt3A or control (PBS with 0.2% serum). Four days after the compound treatment, cells were labeled with [^3^H]-thymidine for four to five hours. Plates were then harvested and counted using TOPCOUNT. Each experiment was carried out in triplicate with error bars representing standard deviation and repeated three times.

### Statistical Analysis

Comparison of cell numbers in cells transfected with GFP, Wnt1 or AR alone, as well as Wnt1 and AR together was conducted using one-tail paired student's t test. P < 0.05 was considered statistically significant.

### Soft Agar growth assay

For analysis of anchorage-independent growth of both treated and untreated parental LNCaP and LNCaP-Flag-AR cells, a soft agarose medium was employed. 1 × 10^4 ^trypsinized cells were resuspended in 1 ml of RPMI containing 10%FBS and 0.3% agarose (type I-A, Sigma) and layered onto a 0.5 ml cushion of 0.6% agarose in RPMI supplemented with 10%FBS in 24-well plates (Costar). The next day, cells were treated with either 10 nM DHT (Sigma) or 100 ng/mL Wnt3A (R&D Systems) in RPMI with 5% charcoal-stripped serum. The medium, with or without treatments, was then renewed by gentle aspiration every 5 days. Cultures were incubated at 37°C in a humidified incubator containing 5% CO_2_, and, after an approximate 4 week growth period, colonies were fixed in the presence of 10% formaldehyde in PBS. Colony growth was assessed by photography, noting size and frequency in a typical field of cells using bright-field microscopy (12.5×). Each experiment was carried out in duplicate. And the experiments have been repeated three times.

### Immunohistochemistry

A tissue microarray was obtained from Asterand containing 4 normal prostate tissue and 9 prostate tumors. This microarray was stained with antibodies against AR (Santa Cruz at a 0.5 ug/ml concentration) and β-Catenin (BD Biosciences, diluted at 1:500), using consecutive slides for the two antibodies. For antigen retrieval, Citra Plus Solution (BioGenex) was used at 95°C for 15 minutes. Slides were stained using the BioGenex i6000 automated staining system. The DAKO Envision Plus kit was used for chromagen detection. As negative controls, isotype mouse IgG1 and rabbit IgG1 were used, and no staining was observed (data not shown).

### Chromatin Immunoprecipitation Assay

LNCaP cells in RPMI with 5% charcoal-stripped serum and 1% Pen-Strep were treated with either 10 nM DHT (Sigma), 100 ng/mL Wnt3A (R&D Systems), or left untreated. After 16 hours the cells were fixed with 2% formaldehyde followed by lysis in 0.2 ml buffer (50 mM Tris pH 8, 10 mM EDTA, 1% SDS, protease inhibitors) per 1 × 10^6 ^cells. After sonication and centrifugation, the supernatant was diluted 1:10 in IP Buffer (16.7 mM Tris-HCl pH 8, 167 mM NaCl, 1.2 mM EDTA, 1.1% Triton ×-100, protease inhibitors) for immunoprecipitation with either anti-AR polyclonal antibody (custom made) or anti-β Catenin mouse monoclonal antibody (Cell Signaling) as well as negative control mouse IgG or rabbit IgG antibodies. No signals were observed in negative controls (data not shown). After overnight incubation at 4°C, protein-DNA complex was then purified with 50 uL of Ultralink Immobilized Protein A/G beads (Pierce) followed by reverse crosslinking. DNA was then isolated using a Qiagen purification kit and eluted in 35 ul volumes. Typically, 2 uL of the purified DNA was used as template in the PCR reactions. The PCR products were analyzed using a Bioanalyzer 2100 (Agilent) and a DNA 1000 kit (Agilent). The amount of PCR product was compared with the input amount to calculate the percentages of input. This experiment was repeated three times and the average of percent input was plotted with error bars representing standard deviation.

The primers used were:

**negative control β-Actin **[57] (No signal was observed in AR IP and β-Catenin IP in Figure 6),

forward 5'-TCCTCCTCTTCCTCAATCTCG-3'

reverse 5'-AAGGCAACTTTCGGAACGG-3'

**PSA Promoter and enhancer **[38]

forward 5'-TCCTGAGTGCTGGTGTCTTAG-3'

reverse 5'-GCCCTATAAAACCTTCATTCCCC-3',

forward 5'-ATGTTCACATTAGTACACCTTGCC-3'

reverse 5'-TCTCAGATCCAGGCTTGCTTACTGTC-3'

**human c-myc and cyclin D1 promoter regions **[37],

forward 5'-GCTCTCCACTTGCCCCTTTTA-3'

reverse 5'-GTTCCCAATTTCTCAGCC-3'

forward 5'-GGGAGGAATTCACCCTGAAA-3'

reverse 5'-CCTGCCCCCAAATTAAGAAAA-3'.

## Authors' contributions

LS designed the experiments, coordinated the assays and the manuscript preparation. In addition, LS performed some of the luciferase reporter assays and the Guava cell count assays. CAR performed some of the luciferase reporter assays, thymidine incorporation assays and soft agar assays. TES performed CHIP assays and AR mutants generation. JSP and QW performed and analyzed IHC study using tissue array. TAL, MMG and RMA supervised and provided guidance and critical input into the study. RMA also participated in the design of the CHIP analysis. All authors have read and approved the final version of the manuscript.
